# An S752D activation loop mutation dynamically primes Muscle-Specific Kinase for activation

**DOI:** 10.1042/BCJ20260159

**Published:** 2026-06-24

**Authors:** Jakob J. Prömer, James W. Murphy, Mark A. Lemmon, Yuko Tsutsui, Ruth Herbst

**Affiliations:** 1 Institute for Specific Prophylaxis and Tropical Medicine, Center for Pathophysiology Infectiology and Immunology, Medical University of Vienna, Vienna, Austria; 2 Yale Cancer Biology Institute, West Haven, CT 06516, U.S.A.; 3 Department of Pharmacology, Yale University School of Medicine, New Haven, CT 06520, U.S.A.

**Keywords:** A-loop autoinhibition, hydrogen-deuterium exchange mass spectrometry, muscle-specific kinase, neuromuscular junction, receptor tyrosine kinases

## Abstract

Muscle-Specific Kinase (MuSK) is a receptor tyrosine kinase essential for neuromuscular junction (NMJ) formation and maintenance, yet its regulation remains poorly understood. Crystallographic studies of wild-type MuSK revealed an autoinhibited conformation with tyrosines in the activation loop (A-loop) anchored within the catalytic cleft to stabilize the closed, inactive conformation. We showed previously that additional phosphorylation of an A-loop serine may ‘prime’ MuSK for activation to sensitize it to ligand(s) in certain settings. Here, we employed crystallography, biochemical assays, and hydrogen–deuterium exchange and mass spectrometry (HDX-MS) to test this hypothesis. We found that introducing a phosphomimetic S752D mutation disrupts autoinhibitory A-loop interactions to increase ATP-binding affinity and catalytic turnover. Using HDX-MS, we further observed that the S752D mutation increases A-loop structural flexibility to relieve autoinhibition. The S752D mutation also stabilizes the juxtamembrane NPXY motif region, a docking site for the adaptor Dok7, possibly priming MuSK for downstream signaling. Together, these findings reveal dynamic transitions that underlie relief of MuSK autoinhibition and provide a mechanistic framework for understanding MuSK activation at the NMJ.

## Introduction

**Figure 1:**
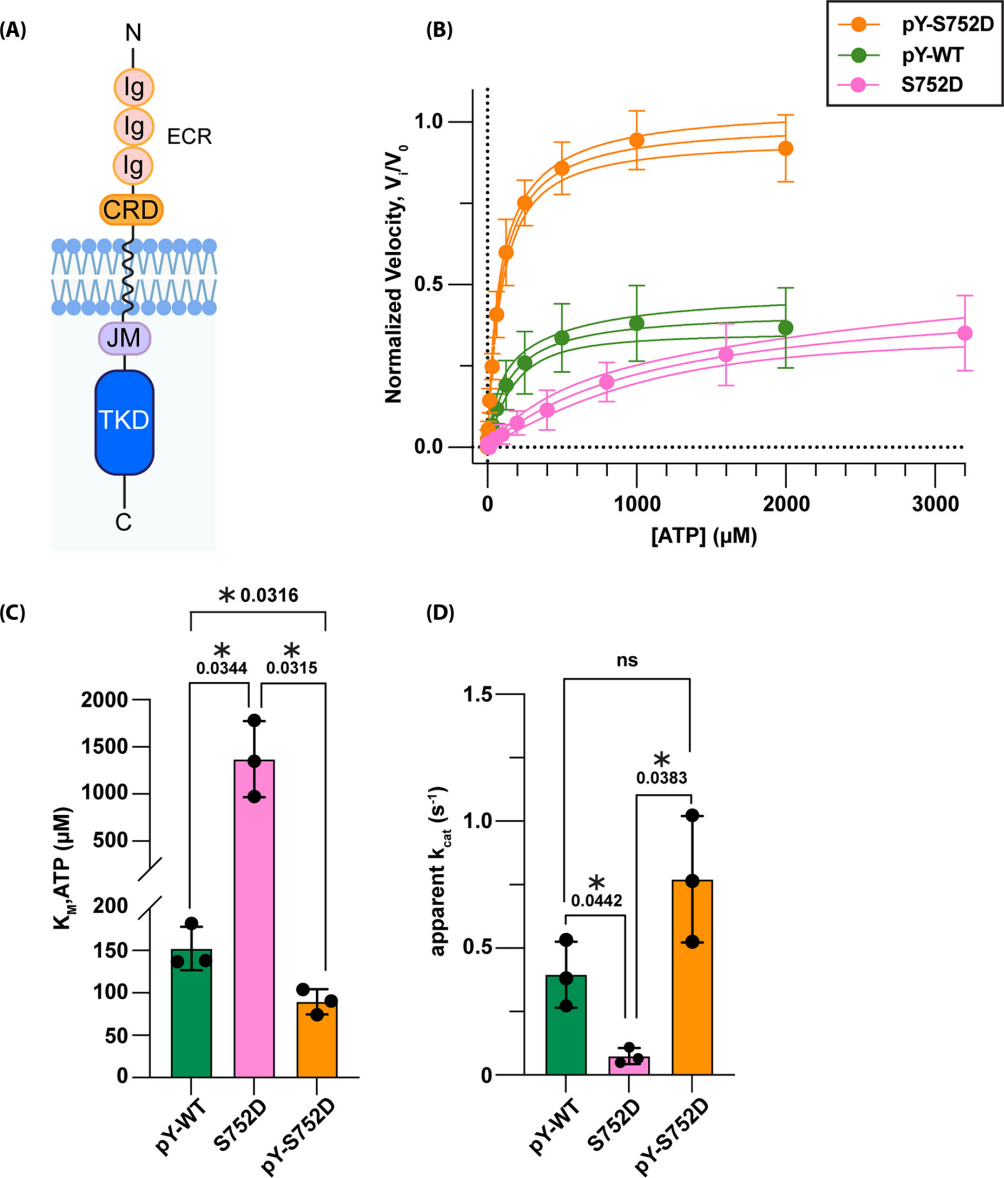
Effects of phosphorylation on MuSK activity (**A**) The domain architecture of full-length MuSK, showing the three extracellular IgG-like domains and Frizzled-like CRD as well as the intracellular JM and TKD. Human MuSK proteins used in the present study contain the intracellular JM region plus TKD (residues 530–869). (**B**) Michaelis–Menten (MM) plots for unphosphorylated MuSK^S752D^ TKD (pink), *in vitro* autophosphorylated MuSK^WT^ TKD (pY-WT, green), and autophosphorylated MuSK^S752D^ TKD (pY-S752D, orange). Normalized mean initial velocity (V_i_/V_0_) values were plotted against ATP concentration and fit to the MM equation to obtain *K*_M, ATP_. (**C**) Bar graph of *K*_M, ATP_ and (**D**) apparent *k*_cat_ obtained from the MM plot for the different protein preparations, as described in the ‘Methods’ section. In panels (C,D), *P*-value <0.05 (*P<0.05) from an unpaired two-sided Welch’s *t*-test are indicated. Values represent the mean ± standard deviation of three independent protein preparation (*n* = 3), with three technical repeat experiments for each protein species.

Muscle-Specific Kinase (MuSK) is a receptor tyrosine kinase (RTK) with an extracellular region (ECR) that contains three immunoglobulin G-like (IgG-like) and a Frizzled-like cysteine-rich domain (CRD). The ECR is connected by a single-pass transmembrane segment and an intracellular juxtamembrane (JM) to the tyrosine kinase domain (TKD) (Figure 1A) [1,2]. MuSK plays an indispensable role in postsynaptic differentiation and the formation as well as maintenance of neuromuscular junctions (NMJs): MuSK-null mice fail to form NMJs, and reduced MuSK expression in adult muscle causes synaptic disassembly [1–3]. At the NMJ, MuSK forms a complex with its co-receptor LRP4 and the extracellular ligand Agrin, which binds LRP4 to activate MuSK [2,4,5]. Upon MuSK activation, the intracellular adaptor Dok-7 binds through its phosphotyrosine-binding (PTB) domain to the tyrosine-phosphorylated conserved NPXY motif in MuSK’s JM region [6–8]. Formation of the Agrin–LRP4–MuSK signaling complex drives clustering of acetylcholine receptors (AChRs) at the postsynaptic membrane, a process that is essential for efficient synaptic transmission between motor neurons and muscle fibers [1,2,9]. Germline mutations in MuSK compromise the functional integrity of the NMJ, resulting in congenital myasthenic syndromes (CMS) that manifest as fatigable muscle weakness from early life [2,10].

Like the TKDs of other RTKs, including insulin receptor kinase (IRK), full activation of the MuSK TKD requires autophosphorylation of three tyrosines in a YxxxYY motif within its activation loop (A-loop). This phosphorylation is facilitated by Agrin binding to LRP4, leading to MuSK dimerization and activation of its TKD at the NMJ [2,11]. Our previous work on MuSK signaling further identified phosphorylation at S751 in the **Y**S*AD**YY** A-loop sequence within MuSK upon Agrin stimulation (mouse and rat S751 is S752 in human MuSK) [12,13]. We proposed that this serine phosphorylation event may play an important role in promoting MuSK activation in the correct contexts. Indeed, myotubes expressing (phosphomimetic) S751D MuSK (MuSK^S751D^) formed larger and more stable AChR clusters upon Agrin stimulation than cells expressing wild-type (WT) MuSK (MuSK^WT^) [12]. Moreover, MuSK^S751D^ activation was found to be significantly enhanced in response to sub-threshold levels of Agrin compared with MuSK^WT^ [12]. These findings argue that A-loop serine phosphorylation adds an additional regulatory layer to MuSK activation beyond canonical tyrosine autophosphorylation in its A-loop YxxxYY motif.

Structural studies of the rat MuSK TKD showed how it is autoinhibited by intramolecular A-loop interactions, with tyrosines of the YxxYY motif occluding substrate binding sites in the same manner as seen in IRK [14,15]. How MuSK relieves these inhibitory contacts to transition into its active state remains unknown, however. Given that A-loop regulatory mechanisms vary greatly across kinases [16–18], defining how MuSK overcomes autoinhibition is crucial to understanding its specific mechanisms of activation during synaptogenesis.

We addressed these questions using a combination of biochemical, structural, and biophysical approaches to examine conformations and dynamics of the human WT and S752D-mutated MuSK TKD. Our studies reveal how A-loop tyrosine autophosphorylation modulates MuSK catalytic activity and how this effect is promoted by the S752D mutation—providing a structural framework for understanding key aspects of MuSK regulation at the NMJ.

## Results

### S752D mutation synergistically enhances MuSK autophosphorylation and kinase activity

We expressed and purified the soluble intracellular regions of both WT and S752D-mutated human MuSK comprising the JM plus TKD, containing residues 530–869 (Figure 1A and Supplementary Figure S1). We first determined steady-state kinetic parameters for kinase activity of each TKD protein using a quantitative fluorometric peptide assay (Figure 1B–D, Supplementary Figure S2, and Table 1), following our previous studies with other TKDs [19]. Earlier studies of unphosphorylated rat MuSK^WT^ TKD reported a high Michaelis constant for ATP (*K*_M, ATP_) of 3.4 mM [15]. Consistent with this, we found that the activity of unphosphorylated human MuSK^WT^ TKD was not saturated at an ATP concentration of 3.2 mM and remained low (Table 1)—preventing us from determining a reliable *K*_M_,_ATP_ for this protein. By contrast, we were able to measure *K*_M,ATP_ for unphosphorylated human MuSK^S752D^ TKD, which returned a value of 1.4 mM (Supplementary Figure S2B and Table 1), with clearly detectable kinase activity (*k*_cat_ = 0.075 s^−1^). Thus, the S752D mutation appears to enhance substrate binding and kinase activity even in the absence of A-loop tyrosine autophosphoryation.

**Table 1:** Steady-state kinetic parameters of WT and S752D MuSK TKDs

MuSK	*K*_M, ATP_ (μM)	*K*_M, peptide_ (μM)	Apparent *k*_cat_ (s^−1^)	*k*_cat_/*K*_M, ATP_ (μM^−1^s^−1^)	*k*_cat_/*K*_M, peptide_ (μM^−1^s^−1^)
**Human**					
WT	n.d.	815	0.001	n.d.	7.0 × 10^−7^
WT, pY	152 ± 25.7	437.3 ± 132.0	0.395 ± 0.130	2.7 × 10^−3^	0.94 × 10^−3^
S752D	1369 ± 404	273.7 ± 89.99	0.075 ± 0.032	6.6 × 10^−5^	1.6 × 10^−4^
S752D, pY	89.5 ± 8.6	633.5 ± 382.3	0.771 ± 0.249	0.0087	0.0030
**Rat** ^a^					
WT	3400	1580	0.0078	2.2 × 10^−6^	4.9 × 10^−6^

Values ±standard deviation represent average of three independent biological repeat experiments (*n* = 3) with three technical repeats/n.

a Data from [15]

Activation of MuSK, like other TKDs such as IRK and the fibroblast growth factor receptor, requires tyrosine autophosphorylation within the A-loop [20]. To examine the functional impact of A-loop tyrosine autophosphorylation, we carried out *in vitro* autophosphorylation of MuSK^WT^ and MuSK^S752D^ and assessed their degrees of phosphorylation using intact mass analysis prior to assaying activity (Supplementary Figure S3 and Table 2). The MuSK TKD contains six known tyrosine autophosphorylation sites, three of which are in the YxxxYY motif located within the A-loop [21]. These six tyrosine autophosphophorylation sites, out of the 19 tyrosines in the TKD, are associated with kinase regulation and downstream signaling [13,15]. *In vitro* autophosphorylation of both MuSK^WT^ and MuSK^S752D^ produced heterogeneous phosphoforms, containing three to six phosphate groups, without detectable residual unphosphorylated protein (Supplementary Figure S3 and Table 2). Notably, all six available autophosphorylation sites were phosphorylated to some extent in MuSK^S752D^, whereas MuSK^WT^ did not reach full phosphorylation under the same conditions (Supplementary Figure S3 and Table 2). Activity assays with autophosphorylated MuSK^S752D^ TKD revealed a *K*_M,ATP_ of 89.5 μM and apparent *k*_cat_ of 0.77 s^−1^, suggesting that autophosphorylation simultaneously increases ATP-binding affinity by ∼15-fold and increases catalytic turnover by ∼10-fold relative to unphosphorylated MuSK^S752D^ (Table 1). Moreover, with A-loop tyrosines phosphorylated, the S752D mutation reduced *K*_M,ATP_ by ∼2-fold compared with autophosphorylated MuSK^WT^ and increased apparent *k*_cat_ by around 2-fold (Figure 1C,D and Table 1).

**Table 2:** Intact mass analysis of unphosphorylated and autophosphorylated (pY) MuSK^WT^ and MuSK^S752D^

#pY	WT			S752D		
	Expected MW (Da)	Observed MW (Da)	Δmass (Da)	Expected MW (Da)	Observed MW (Da)	Δmass (Da)
0	39 886	39 225.7 ± 0.47	38	39 914	39 954.1 ± 0.40	40
1	Not observed	Not observed	–	39 994	40 034.3 ± 0.00	40
*3*	*40 126*	*40 166.6 ± 0.76*	*40*	*Not observed*	*Not observed*	*–*
*4*	*40 206*	*40 246.8 ± 0.71*	*40*	*40 234*	*40 274.1 ± 0.26*	*40*
*5*	*40 286*	*40 326.8 ± 0.53*	*40*	*40 314*	*40 353.8 ± 0.05*	*40*
*6*	*Not observed*	*Not observed*	*40*	*40 394*	*40 434.3 ± 0.30*	*40*

The italic data are intact mass values of the autophosphorylated samples (see the ‘Methods’ section). The mass difference (Δmass) of 38–40 Da is due to the N-terminal acetylation or Na^+^/NH^+^ adduct formation. The error values are standard deviation calculated from at least three biological repeats (n ≥ 3).

Together, these results demonstrate that the S752D mutation enhances ATP-binding affinity of the MuSK TKD in the absence of autophosphorylation and/or promotes MuSK tyrosine autophosphorylation. In addition, the S752D mutation enhances MuSK catalytic activity *in vitro* (Figure 1C,D and Table 1), which correlates with the extent of autophosphorylation (Supplementary Figure S3 and Table 2).

### Crystal structure comparison of WT and S752D MuSK TKDs

**Figure 2:**
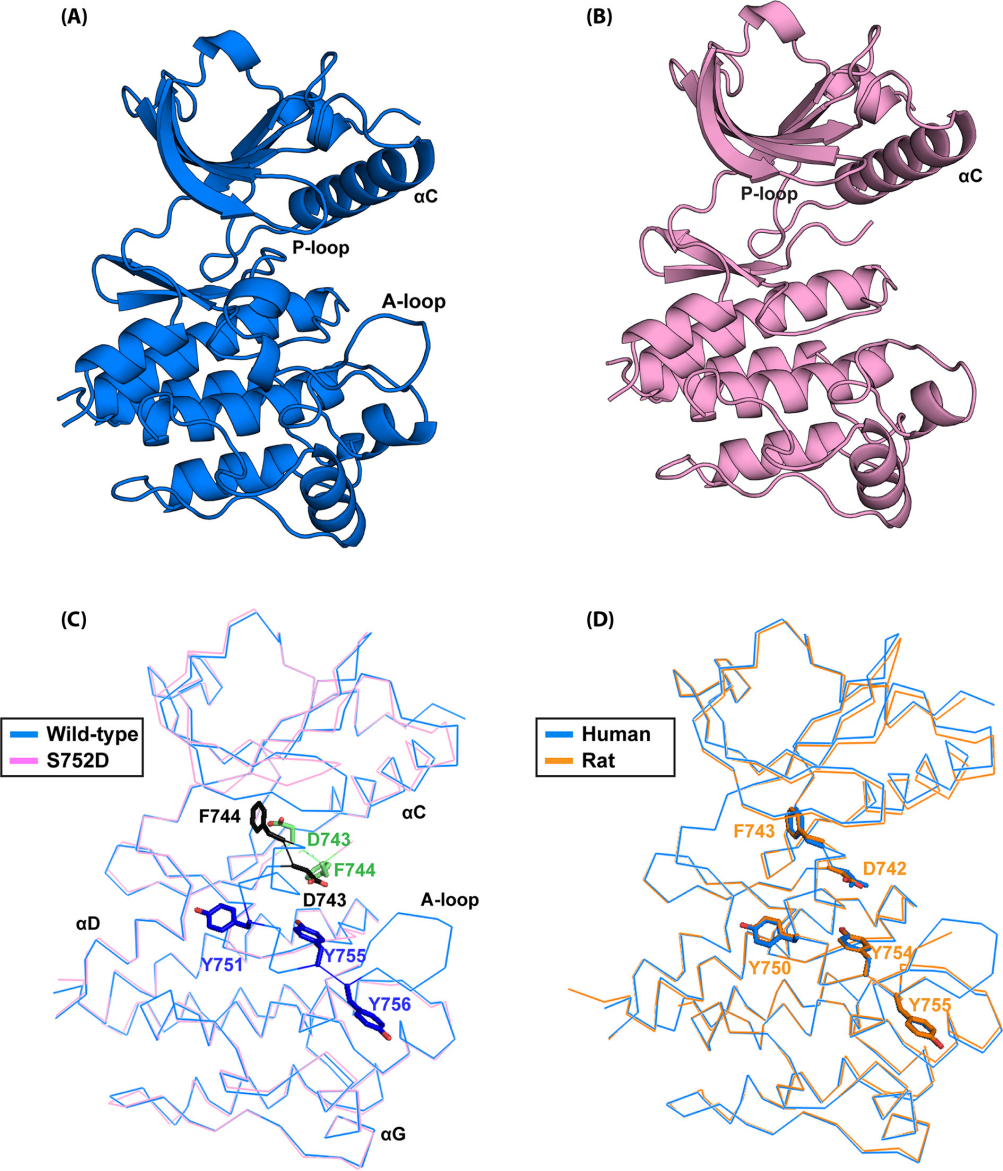
Crystal structures of human MuSK TKD with DFG-out and -in conformations (**A,B**) Crystal structures of MuSK^WT^ (**A**) and MuSK^S752D^ (**B**) TKDs at 2.2 and 2.6 Å, respectively, are shown in cartoon representation. For crystallographic statistics, see Table 3. (**C**) Overlay of crystal structures of MuSK^WT^ (light blue) and MuSK^S752D^ TKDs. D743 and F744 of the conserved DFG motif in MuSK^WT^ and MuSK^S752D^ are shown as black and light green sticks, respectively. Y751, Y755, and Y756 of the YxxxYY motif in the MuSK^WT^ A-loop are shown as blue sticks. The corresponding motif in MuSK^S752D^ is not shown due to poorly defined electron density. (**D**) Overlay of human MuSK^WT^ (light blue) and rat MuSK^WT^ (PDB ID: 1LUF, orange) TKDs is shown. D742 and F743 of the conserved DFG motif and the YxxxY motif in rat TKD are shown as orange sticks with rat MuSK residue numbers, as indicated. Blue sticks show the corresponding residues in human MuSK^WT^.

The activating effect of S752D prompted us to determine a crystal structure of MuSK TKD harboring this mutation. An earlier crystal structure of WT rat MuSK TKD in an inactive conformation revealed an autoinhibition mechanism in which the DFG phenylalanine occupies the position normally taken by the adenine ring of ATP, displacing the conserved DFG aspartate from its catalytically competent position. The YxxxYY tyrosines also occlude the substrate binding sites in this autoinhibited structure [15]. To understand how introducing an additional negative charge in the A-loop may contribute to MuSK regulation, we determined new crystal structures of human MuSK^WT^ and MuSK^S752D^ TKDs at 2.2 Å and 2.6 Å, respectively (Figure 2A–C and Table 3). Both structures adopt the canonical bilobed kinase fold typical of protein kinases. In both structures, electron density corresponding to part of the JM region (residues 530–559 for WT and 530–561 for S752D) and the serine-rich connecting loop between ⍺D and ⍺E (residues 673–695) are missing. The overall structures of human MuSK^WT^ and MuSK^S752D^ are highly similar to the rat MuSK^WT^ TKD, with backbone RMSDs of 0.34 and 0.62 Å, respectively (Figure 2D).

**Table 3:** Crystallization conditions, data collection, and refinement statistics

Protein	MuSK^WT^	MuSK^S752D^
PBD ID	9SN0	9SNU
Crystallization conditions	4 mg/ml protein, 0.1 M HEPES (pH 7.0), 0.5 M (NH_4_)_2_SO_4_, 0.1 M NaSCN, 1.5 mM TCEP, 2% glycerol 19°C	4 mg/ml protein, 0.1 M HEPES (pH 7.0), 20% PEG 3350, 0.1 M NaSCN, 1.5 mM TCEP
**Data collection** ^a^		
Beamline	FMX (17-ID-2)	(AMX 17-ID-1)
Date of collection	March 3^rd^ 2025	June 5^th^ 2025
Wavelength (Å)	0.979330	0.919696
Space group	*I 4*	*I 4*
Cell dimensions		
a, b, c (Å)	148.378, 148.378, 39.345	149.352 149.352 39.435
α, β, γ (°)	90, 90, 90	90 90 90
Resolution (Å)	46.92–2.2 (2.3–2.2)	47.23–2.62 (2.88–2.62)
Completeness (%)	99.9 (99.5)	99.7 (98.1)
Redundancy	7.3 (7.2)	4.1 (4.0)
R_sym_ (%)	13.5 (196.7)	8.0 (77.4)
I/σ	12 (2.1)	11.2 (1.7)
CC^1/2^	0.994 (0.640)	0.997 (0.522)
**Refinement**		
Number of reflections	22004 (2544)	13.387 (3260)
R_work_/R_free_ (%)	21.1 / 23.1	21.1 / 24.0
Number of atoms		
Protein	2209	2076
Ligands	5	0
Water	125	64
Average B factor (Å)		
Protein	42.93	69.75
Ligands	62.16	-
Water	45.63	62.53
Ramachandran favored (%)	97.11	96.88
Ramachandran allowed (%)	2.89	3.12
Ramachandran outliers (%)	0.0	0.0
Bond length rmsd (Å)	0.004	0.002
Bond angle rmsd (Å)	0.65	0.5

In the human MuSK^WT^ TKD structure, the side chain F744 in the DFG motif superimposes with the corresponding F743 in the inactive rat structure (Figure 2D), and the A-loop forms a short helical turn characteristic of an inactive TKD conformation, placing the YxxxYY tyrosines in the same positions as seen in the autoinhibited rat MuSK^WT^ TKD (Figure 2D). By contrast, in the MuSK^S752D^ TKD structure, the F744 side chain instead adopts a position typical of that seen for active DFG-in TKD conformations (green in Figure 2C), although it does not fully superimpose with the DFG phenylalanine in a canonical active-state structure seen for IRK (Supplementary Figure S4A).

Additional structural features also distinguish the human MuSK^S752D^ TKD from ‘fully active’ TKDs. Whereas the P-loop folds over the ATP binding pocket in fully activated IRK with phosphorylated A-loop tyrosines, the P-loop is displaced upward in MuSK^S752D^ (Supplementary Figure S4A). Similarly, αC remains in an ‘out’ position in unphosphorylated MuSK^S752D^, contrasting with the αC-in position characteristic of fully active IRK (Supplementary Figure S4A). The β3-αC salt bridge, a hallmark indicator of active kinases, also differs between MuSK and IRK (Supplementary Figure S4B,C). In IRK, the distance between the β3 lysine and the αC glutamate side chain shows a pronounced shift between inactive and active structures. The β3 lysine ζ nitrogen goes from 6.0 Å away from Oε1 of the αC glutamate (inactive) to 4.5 Å (active), and the lysine Nζ goes from 8.0 Å away from the αC glutamate Oε2 (inactive) to 4.4 Å (active) (Supplementary Figure S4B,C). In contrast, the corresponding distances in MuSK change only slightly between the WT and S752D structures (K609Nζ and E626Oε1: 4.8 Å (WT, inactive) → 4.6 Å (S752D); K609Nζ and E626Oε2: 4.0 Å (WT, inactive) → 4.3 Å (S752D)) (Supplementary Figures S4B,C). Together with the other observations summarized above, these findings suggest that the MuSK^S752D^ TKD acquires some features of an active kinase conformation even in the absence of A-loop tyrosine phosphorylation but is not in a fully active conformation until it is tyrosine phosphorylated. In other words, the S752D mutation appears intriguingly to stabilize a ‘pre-activated’ conformation that may be ‘poised’ for full activation.

### S752D relieves autoinhibition by breaking the key A-loop hydrogen bonds and π–π stacking

**Figure 3:**
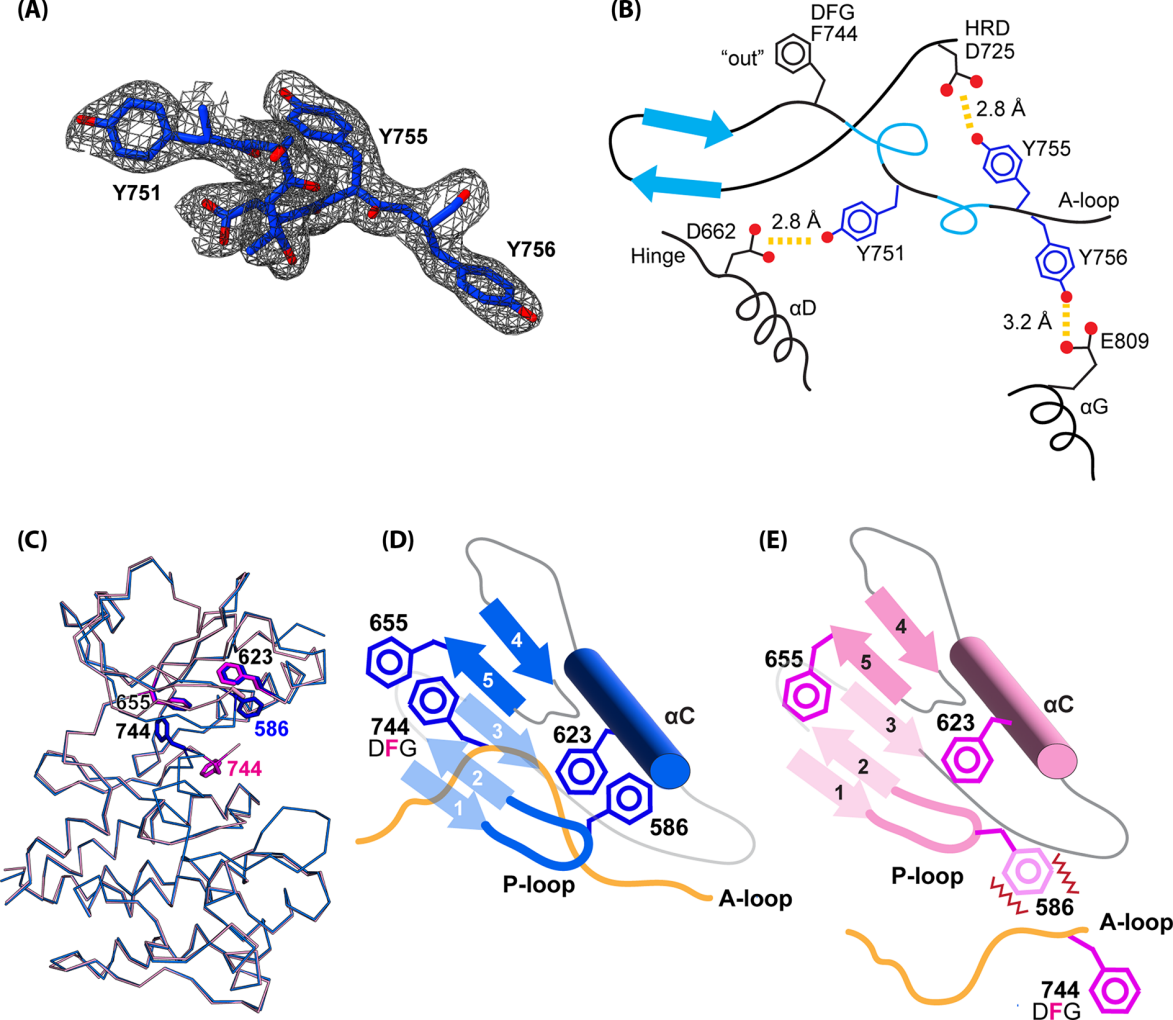
S752D mutation breaks π–π stack interactions to release autoinhibition (**A**) The 2Fo-Fc map of the human MuSK^WT^ A-loop region containing phosphosites, Y751, Y755, and Y756, contoured at 1σ is shown as black mesh. Oxygen atoms are colored red. (**B**) cartoon of A-loop anchoring interactions. The side chain of F744 in the conserved DFG motif is colored black. Phosphosite tyrosine side chains (Y751, Y755, and Y756) are colored blue, with oxygen atoms as red spheres. Hydrogen bond distances between each phosphosite tyrosine hydroxyl and an interacting residue are indicated (dotted orange lines). (**C**) Overlay of MuSK^WT^ (blue) and MuSK^S752D^ (pink). Phenylalanine side chains involved in π–π stacking interactions are shown as blue (WT) and magenta (S752D) sticks. Poorly defined electron density for F586 in the MuSK^S752D^ P-loop indicates structural flexibility in this region, supported by HDX-MS data. Phenylalanine side chains involved in π–π stacking interactions in MuSK^WT^ (**D**) and MuSK^S752D^ (**E**) are shown in blue and magenta, respectively, with residue numbers indicated. The side chain ring of F586 in the MuSK^S752D^ P-loop is shown in panel (E) as a transparent magenta cartoon highlighted with red wavy lines due to its ambiguous electron density.

Unambiguous A-loop electron density in the human MuSK^WT^ structure (Figure 3A) revealed key autoinhibitory interactions involving the conserved YxxxYY tyrosines in the A-loop. The Y751 side chain is within hydrogen-bonding distance of the side chain of D662 in the hinge region, N-terminal to αD (Figure 3B). The Y755 side chain forms a predicted hydrogen bond with the catalytic base aspartate (D725) in the conserved ‘HRD’ motif (Figure 3B). The Y756 side chain is within hydrogen bonding distance of the side chain of E809 in αG (Figure 3B). As with most other tyrosine kinases that have the YxxxYY motif in their A-loop [14,15,22], these interactions appear to stabilize the autoinhibited A-loop conformation characteristic of the inactive conformation, which must be disrupted in order for these tyrosines to become accessible for phosphorylation in the activated receptor dimer [23,24]. In our MuSK^S752D^ structure, we were not able to trace the complete A-loop in the electron density map, arguing that the S752D mutation increases flexibility in this region—in a way that may weaken the interactions mentioned above and ‘sensitize’ MuSK to activation. In an A-loop region with increased conformational dynamics, these key autoinhibitory interactions listed above would be expected to be compromised on average, which might be expected to correlate with enhanced activity—as we saw biochemically (Figure 1C,D and Table 1).

Another interaction altered in the MuSK^S752D^ crystal structure relative to MuSK^WT^ is the π-stacking interaction between F586 in the glycine-rich P-loop and F623 in αC (Figure 3C,D). Disrupting this interaction in MuSK^S752D^ (Figure 3E) appears to ‘free’ up αC (which contains F623) to allow a transition to an active-like conformation. Moreover, the S752D mutation appears to disrupt a π-stacking interaction seen in MuSK^WT^ between F744 of the TKD’s conserved DFG motif and F655 in β5 (Figure 3C,D), which contributes to the interface between the N- and C-lobes and restrains the ‘out’ (inactive) configuration of the MuSK^WT^ DFG motif (Figure 3C,D). In the MuSK^752D^ TKD, F744 of the DFG motif points in a quite different direction (Figure 3C,E) so that it resembles the position of the DFG phenylalanine in active IRK (Supplementary Figure S4A). Together, these structural changes further suggest that the S752D mutation reduces restraints on the position of αC in MuSK and also ‘loosens’ the N-lobe/C-lobe interface to reduce restraints on the DFG motif—‘priming’ MuSK for full activation. These rearrangements also alter the ATP binding pocket in a way that would be anticipated to increase ATP accessibility. Indeed, these structural differences between MuSK^WT^ and MuSK^S752D^ correlate well with the observed ATP affinities and kinase activities (Figure 1C,D and Table 1).

### S752D impacts the dynamics of key functional regions, including the JM and A-loop

**Figure 4:**
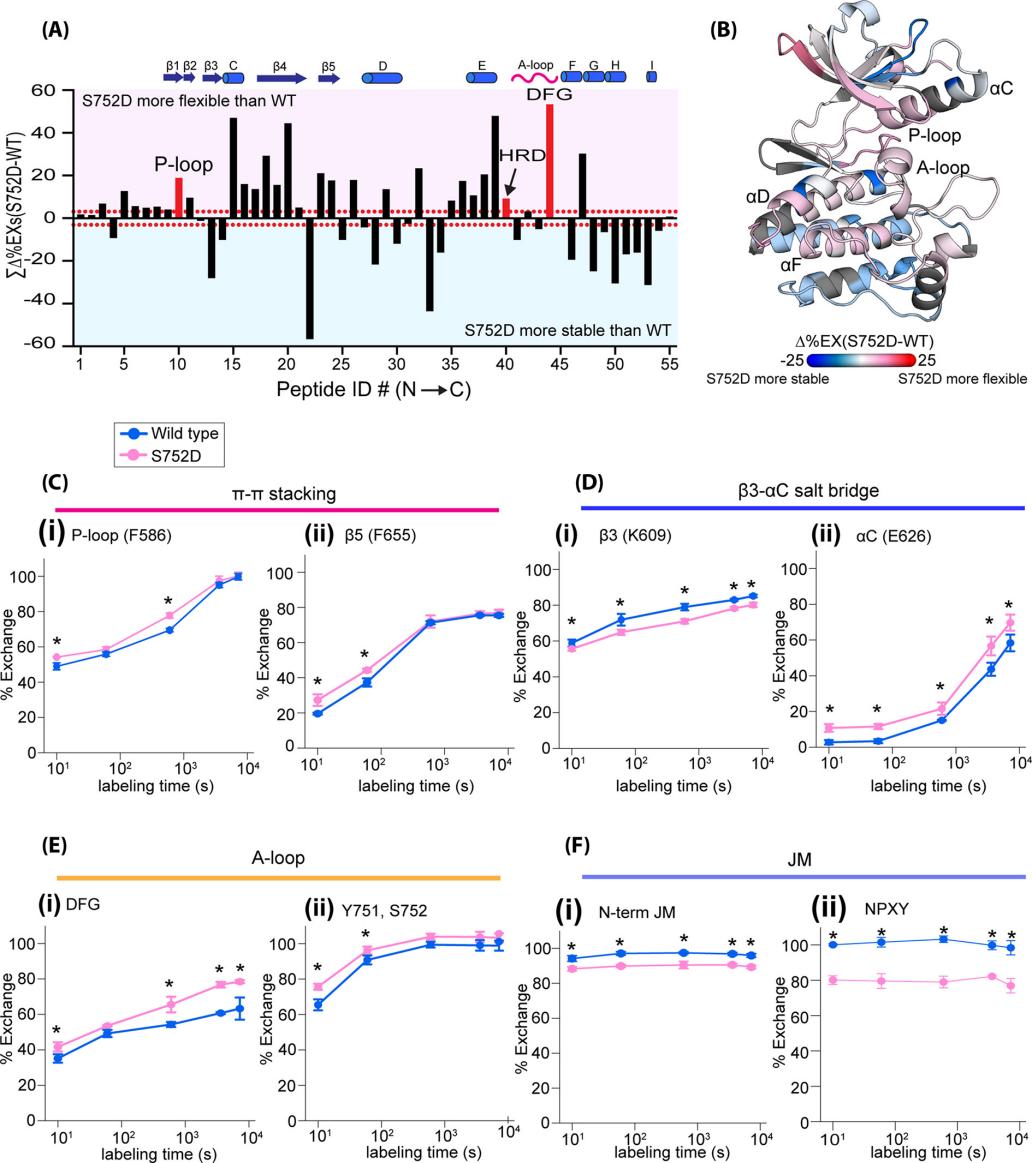
The S752D mutation increases A-loop structural flexibility (**A**) Butterfly plot, showing summed percent exchange differences between MuSK^S752D^ and MuSK^WT^ (ΣΔ%EXs) across all labeling time points, with peptides shown as black bars from N- to C-terminus. The % exchange values of the same peptides between MuSK^WT^ and MuSK^S752D^ are compared. The *X*-axis shows peptide ID numbers; residue range and amino acid sequence of each peptide are listed in Supplementary Table S1. Red bars show known functionally important regions. Red-dotted lines indicate the 95% confidence threshold (ΣΔ%EXs = 3.1% corresponding to ΣΔDuptake = 0.31Da), as calculated according to [39]. The black bars exceeding this threshold denote regions with statistically significant dynamic changes upon S752D mutation. Negative and positive Δ%EXs values indicate regions that are more stable or more flexible, respectively, in S752D relative to WT. Secondary structure elements are indicated above the plot. (**B**) Δ%EX (S752D-WT) values at 600 s labeling time point mapped onto the crystal structure of human MuSK TKD. The same peptides from MuSK^WT^ and MuSK^S752D^ were used to calculate Δ%EX. For structure maps at other labeling timepoints, see Supplementary Figure S8. Blue and red regions show increased stability and flexibility, respectively, in MuSK^S752D^. Dark gray signifies residues lacking coverage. Percent exchange of peptides involved in (**C**) π–π stacking interactions: (i) residues 578–586 (F586 in P-loop) and (ii) residues 655–663 (F655 in β5); (**D**) β3-αC salt bridge: (i) residues 608–619 and (ii) residues 624–630. Percent exchange of peptides containing (**E**) DFG: (i) residues 740–746, (ii) Y751 and S752: residues 744–754; (**F**) JM region: (i) residues 536–543, (ii) residues 544–557 containing the NPXY motif; The gray percent exchange data points in panels (C–F) indicate time points at which no statistically significant structural change between MuSK^WT^ and MuSK^S752D^, as calculated based on [39]. All percent exchange data are the average of three biological repeat experiments (*n* = 3) with three technical repeats for each *n*. See Table 4 for HDX-MS data statistics. Asterisks on the top of each data point show statistically significant Δ%EX based on the statistical analysis by Houde et al. [39].

Differences in electron density definition in the A-loop and elsewhere when comparing the MuSK^WT^ and MuSK^S752D^ maps suggest that—beyond the static structural changes outlined above—there are likely to be differences in dynamics and conformational ensembles of the two variants that contribute to differences in their activities. The extent of these differences may be masked by the overall RMSD of only 0.37 Å between the two static structures when the A-loop is excluded. To probe the effects of the S752D mutation on dynamics of the MuSK TKD structure, we employed hydrogen–deuterium exchange and mass spectrometry (HDX-MS). Intact purified human MuSK^WT^ and MuSK^S752D^ were deuterium-labeled for different times and then protease-digested for peptide MS analysis to identify structurally more flexible and rigid regions in the respective TKD native states (Figure 4A,B, Supplementary Figures S5–S8, and Supplementary Table S1). Regions with greater conformational flexibility show higher deuterium uptake (% exchange) than more rigid regions over a given exchange time. Accordingly, differences in percent deuterium exchange between MuSK^WT^ and MuSK^S752D^ (Δ%EX) report alterations in flexibility or stability when the S752D mutation is introduced (Figure 4A,B and Supplementary Figures S7 and S8). Table 4 shows HDX-MS data statistics. In Figure 4A, positive Δ%EX values indicate regions that become more flexible (or destabilized) with the S752D mutation, whereas negative Δ%EX values indicate regions that become more stabilized. Figure 4B and Supplementary Figure S8 visualize these dynamic changes mapped onto the MuSK TKD structures.

**Table 4:** HDX-MS data statistics

Data set	MuSK^WT^	MuSK^S752D^
HDX reaction details	20 mM HEPES, 100 mM NaCl, pD7.4, 25°C	
HDX time course (s)	10, 60, 600, 3600, 7200	
HDX control samples	20 mM HEPES, 100 mM NaCl, 8 M urea-d4, pD 7.4	
Back-exchange (%)	41	42
# of peptides	103	72
Sequence coverage (%)^*^	99%	98%
Average peptide length/redundancy	11.2/3.8	11.3/2.6
Replicates (biological and technical)	*n* = 3 (3 tech repeats/*n*)	*n* = 3 (3 tech repeats/*n*)
Repeatability (Ave σ)^**^	± 0.091 Da (1.6%)	± 0.098 Da (1.9%)
Significant differences in HDX (Δ %EX S752D-WT)^***^	≥0.31 Da (3.1%), 95% CI	

* Excluding the N-terminal 6× His sequence.

** Average standard deviation (SD) calculated based on the SD of three biological experiments (3 technical repeats/*n* biological), the number in parentheses shows SD in percentage.

*** The significant difference in Δ %EX (S752D-WT) calculated according to [39].

The S752D mutation impacts global structural dynamics of the MuSK TKD, increasing flexibility in several functionally important regions, including the glycine-rich-P-loop and regions containing the HRD and DFG motifs (Figure 4A and Supplementary Figure S7). Increased flexibility is also observed towards the C-terminus of αC and in part of αE (Figure 4A,B), indicating that the A-loop S752D mutation exerts long-range allosteric effects across the kinase domain. These mutation-induced changes occur in a time-dependent manner, reflecting region-specific free energy differences for accessing exchange-competent conformations.

Regions involved in π–π stacking interactions undergo statistically significant changes at early labeling times, with increased exchange observed at 10 s and 10 min in the P-loop and at 10 and 60 s in the F655-containing β5 strand (Figure 4C). By contrast, regions encompassing the β3-αC salt bridge show altered dynamics across a broad temporal range, from seconds to hours (Figure 4D), with the S752D mutation reducing exchange in the β3 peptide but increasing exchange in the αC peptide. Within the A-loop, dynamic responses are distinct: a seven-residue segment encompassing the DFG motif displays increased exchange in MuSK^S752D^ at early (10 s), intermediate (10 min), and late (hour) time points (Figure 4E(i)), whereas an adjacent region containing Y751 and S752 shows significant increases only at early time points (10–60 s) (Figure 4E(ii)). These data may suggest that the A-loop comprises dynamically distinct subregions that respond independently to the S752D mutation. Furthermore, the synchronous early-time alterations observed in the P-loop and the β5 strand may point to a coordinated allosteric network across these regulatory elements.

Beyond effects within the TKD core, HDX-MS analysis also reveals dynamic changes that may ‘prime’ MuSK for interactions with downstream signaling proteins. Although the JM region is not resolved in the crystal structures (Figure 2A,B), HDX-MS suggested significant dynamic differences between this region of MuSK^WT^ and MuSK^S752D^ across all labeling times, with reduced exchange in the mutated variant (Figure 4F). The fact that the NPXY motif, known to be a docking site for the PTB domain of Dok7 [6], showed reduced deuterium uptake in MuSK^S752D^ (Figure 4F(ii)) suggests that its local stability is enhanced. This observation suggests that the S752D mutation may promote formation of a more ordered JM scaffold, potentially pre-organizing MuSK for downstream effector binding.

Taken together with our crystal structure analysis, these HDX-MS results demonstrate that the S752D mutation increases flexibility within the P-loop, αC, and DFG motif while stabilizing the NPXY-containing JM region that binds effectors—thus simultaneously weakening autoinhibitory interactions while potentially priming MuSK for full activation and downstream signaling.

## Discussion

MuSK is a central regulator of NMJ formation, yet the detailed molecular mechanisms that couple its kinase activation to downstream signaling have remained poorly defined. In the present study, we combined enzymology, X-ray crystallography, and HDX-MS to ask how regulation of MuSK’s tyrosine kinase activity is influenced by a recently discovered serine phosphorylation event in its A-loop [12].

Steady-state kinetic analysis demonstrates that S752D mutation and A-loop autophosphorylation synergistically enhance catalytic activity by increasing both ATP affinity and turnover. Whereas the activity of unphosphorylated MuSK^WT^ was too low to be measured reliably, significant activity was seen for MuSK^S752D^ even in the absence of A-loop tyrosine phosphorylation (Figure 1C,D and Table 1). Similarly, although A-loop tyrosine phosphorylation alone conferred substantially enhanced activity, this was potentiated by the S752D mutation (Figure 1C,D and Tables 1 and 2). We therefore propose that the S752D mutation in MuSK lowers the energetic barrier for activation of this TKD. In this model, S752D phosphorylation enhances MuSK responsiveness to subsequent tyrosine phosphorylation *in vivo* rather than acting as a simple binary on/off switch.

We previously showed that CAMK2 kinases can phosphorylate a triple-tyrosine-phosphorylated MuSK A-loop peptide *in vitro* and confirmed this in cultured cells [13]. Searching the Kinase Library (https://kinase-library.mit.edu/home; search term: GLSRNIyS*ADyyKADGL) yielded 25 additional potential candidate matches, besides CaMK2 isoforms (α, β, and γ), including casein kinases (CKs), yet another kinase (YANK), G protein-coupled receptor kinases (GRKs), and glycogen synthase kinase (GSK). While YANK was not in our profiling panel, none of the other predicted candidates (CK2α, GRK2, or GSK) showed significant peptide phosphorylation under our experimental conditions [13]. Notably, S752 phosphorylation was still observed in CAMK2β knockout muscle cells as well as *CAMK2β^−/−^* mice, suggesting compensation by other CAMK2 isoforms or the involvement of a yet unidentified kinase [13].

We also investigated the structural basis of the mechanism of priming by S752 phosphorylation using crystallography and HDX-MS. Our results demonstrate that the native, unliganded MuSK landscape is fundamentally shifted by the phosphomimetic S752D mutation. Structural analyses indicate that MuSK^WT^ employs a similar autoinhibitory mechanism to the IRK family [14], in which conserved A-loop tyrosines obstruct ATP and substrate access in the inactive state (Figure 3). Kinase activation involves disruption of local interactions responsible for this obstruction, which is achieved by autophosphorylation of the A-loop tyrosines. Our crystal structure of MuSK^S752D^ suggests that—prior to tyrosine autophosphorylation—the A-loop tyrosines are more accessible than those in MuSK^WT^, which may allow them to be phosphorylated more readily. We suggest that this difference may effectively ‘prime’ MuSK TKD for activation. Consistent with this ‘priming’ hypothesis, our HDX-MS analysis revealed that the S752D mutation induces pronounced dynamic remodeling throughout the kinase domain (Figure 4 and Supplementary Figures S7 and S8). Increased flexibility is seen in key regions that stabilize the autoinhibited state, including the P-loop (Figure 4C(i)), β5 strand (Figure 4C(ii)), αC helix (Figure 4D(ii)), and the DFG motif (Figure 4E(i))—consistent with the mutation ‘loosening’ the ATP binding pocket and weakening autoinhibitory constraints, while coordinated shifts in the conserved HRD and DFG motifs may further facilitate the transition to an active state. These HDX-MS results suggest that the native state dynamics of MuSK encode intrinsic propensity for autophosphorylation and priming and that the S752D mutation fundamentally remodels the MuSK energy landscape.

Because S752 phosphorylation was observed upon Agrin stimulation of MuSK at NMJs *in vivo* [12], we suggest that the S752D crystal structure (Figure 2B) may represent a phosphorylation-driven ‘pre-activated’ conformation in which the MuSK TKD is dynamically primed for subsequent A-loop phosphorylation and recruitment of downstream signaling partners such as Dok-7 [5,13,25,26]. In support of this model, we also saw that the S752D mutation increases HDX protection in the JM region NPXY motif (Figure 4F(ii)), suggesting possible allosteric communication between the A-loop and JM region to couple catalytic priming with signaling complex assembly.

In summary, our biochemical, structural, and dynamic analyses of human MuSK suggest a mechanism by which MuSK activation is achieved through relief of A-loop-mediated autoinhibition and dynamic priming of the kinase domain. More broadly, these findings highlight the importance of conformational dynamics in RTK regulation and illustrate how dynamic allostery may integrate kinase activation with scaffold assembly. Phosphorylation serves as a central regulatory and feedback mechanism in RTKs, ensuring that kinase activation is tightly controlled, context-dependent, and precisely tuned to signaling demands. The presence of multiple phosphorylation sites and autoinhibitory interactions may provide a mechanism for fine-tuning MuSK activity levels in response to cellular ATP availability and metabolic state through A-loop phosphorylation.

## Methods

### Construction of human MuSK TKD (residues 530–869) expression plasmids

The JM region and TKD (residues 530–869) were amplified from a full-length MuSK expression construct, obtained from the I.M.A.G.E. cDNA library (these residues correspond to 444–783 in gene accession BC109098.1) as a PCR template. An NcoI restriction site and 6x-His-tag at the N-terminus of the protein were also introduced by PCR (forward primer aaaacCATGGCGAATCAGCAGCAGTAACCCTC, reverse primer AAAGGGGGATGTGCTGCAAGGC). A PCR-derived DNA fragment encoding MKKGHHHHHHG-hMuSK residues 530–869 was then cloned into pFastBac1 using NcoI and XhoI. The point mutation S752D was introduced by site-directed mutagenesis (forward primer GAACATCTACgatGCAGACTACTACAAAGCTAATGAAAAC, reverse primer GAACATCTACgcgGCAGACTACTACA) using Q5-site-directed mutagenesis (New England Biolabs). Recombinant baculovirus was generated using the Bac-to-Bac expression system (Invitrogen, Waltham, MA, U.S.A.), as described previously, using the single-step PEI transfection protocol [27].

### Human MuSK TKD expression and purification

Sf9 cells (Expression Systems) at 1.8–2.0 × 10^6^ cells/ml were infected with baculovirus and cultured for 48 h at 27°C. The Sf9 cells were then harvested by centrifugation at 3000×***g*** for 30 min, washed with cold (4°C) phosphate-buffered saline, and resuspended in cold lysis buffer (20 mM Tris pH 8.5, containing 150 mM NaCl, 20 mM imidazole, 1% (w/v) glycerol, 1 mM TCEP), supplemented with cOmplete protease inhibitor cocktail (Roche). The resuspended cells were lysed using an M110P microfluidizer (Microfluidics Inc., Newton, MA, U.S.A.) followed by centrifugation at 20 000×***g*** for 30 min to clarify the cell lysate. The supernatant was filtered through a 0.22-μm bottle-top filter (Millipore) and loaded onto a 5-ml HisTrap HP column (Cytiva) equilibrated in buffer A (20 mM Tris, pH 8.5, containing 150 mM NaCl, 1% (w/v) glycerol, and 1 mM TCEP). The column was washed with 10 column volumes (CV) of 8% (v/v) buffer B (buffer A + 500 mM imidazole, pH 8.5), and proteins were eluted using 30% (v/v) buffer B at 5 ml/min.

Pooled HisTrap fractions were diluted 1:4 (v/v) with anion exchange buffer C (20 mM Tris pH 8.5, containing 1% (w/v) glycerol, 1 mM TCEP) to a final concentration of 37 mM NaCl and loaded onto a 5 ml HiTrap Q FF column (Cytiva). Proteins were eluted using a linear gradient of 3.8% to 30% anion exchange buffer D (20 mM Tris, pH 8.5, containing 1 M NaCl, 1% (w/v) glycerol, and 1 mM TCEP) at 5 ml/min. Anion exchange elution fractions containing MuSK were pooled, concentrated using 15 ml AMICON Ultra centrifugal filters with 30 kDa MWCO (Merck Millipore), and then loaded onto a Superdex 75 10/300 column (Cytiva) equilibrated in buffer A (20 mM Tris pH 8.5, containing 150 mM NaCl, 1% (w/v) glycerol, 1 mM TCEP). Purity of the pooled fractions was verified by 10% SDS–PAGE (Supplementary Figure S1B,D), and fractions containing MuSK were concentrated using a 4-ml AMICON Ultra centrifugal filter (30 kDa MWCO). Protein concentrations were determined by measuring absorbance at 280 nm using a molar extinction coefficient of 50 130 M^−1^cm^−1^.

### Autophosphorylation and intact mass analysis

Purified human MuSK TKD at 1 mg/ml was incubated in a total volume of 450 μl buffer A, containing 10 mM ATP and 20 mM MgCl_2_ at 37°C for 10 min, followed by 110 min incubation at 25°C for autophosphorylation. The phosphorylated sample was run on a Superdex 75 10/300 column (Cytiva) to remove residual ATP and MgCl_2_, followed by intact mass analysis on an Acquity UPLC system with a CSH C4 trap (2.1 × 5 mm, Waters) and an analytical CSH C4 column (2.1 × 100 mm, Waters) interfaced with Xevo G2-XS Q-Tof (Waters, Milford, MA). The columns were equilibrated in 95% solvent A (water + 0.1% formic acid), and the protein was eluted using a step gradient from 5% to 98% B (acetonitrile + 0.1% formic acid) at 0.3 ml/min. The collected MS spectra were analyzed using MassLynx 4.2 MaxEnt1 (Waters) to determine the average molecular weight of autophosphorylated proteins (Supplementary Figure S3 and Table 2).

### Determination of human MuSK crystal structures

Crystals were obtained using the hanging drop vapor diffusion method using reservoir solutions of either 0.1 M HEPES (pH 7.0), containing 0.5 M ammonium sulfate, 0.1 M sodium thiocyanate, 2% (w/v) glycerol, and 1.5 mM TCEP or 0.1 M HEPES (pH 7.0), containing 20% PEG 3350 (w/v), 0.1 M sodium thiocyanate, and 1.5 mM TCEP at a 3:1 (v/v) (protein: reservoir) ratio at 19°C. Crystals appeared within 24 h and were cryo-protected in 25% (w/v) glycerol, 25% (w/v) 1,3-propanediol, 12.5% (w/v) sucrose, and 12.5% (w/v) glucose. Diffraction data were collected on FMX 17-ID-2 (WT MuSK) or AMX 17-ID-1 (S752D MuSK) beamlines at the National Synchrotron Light Source II (Brookhaven National Laboratories). Diffraction data were integrated using X-ray detector software [28,29] and scaled using AIMLESS/Ctruncate in CCP4 [30]. Structures were solved by molecular replacement using a rat MuSK structure (PDB ID: 1LUF) [15] as the search model. Model building was performed using Coot [31] with refinement using Phenix 1.21.2-5419 [32,33]. Structure figures were generated using PyMOL v 3.1.3. (The PyMOL Molecular Graphics System, Version 3.0 Schrödinger, LLC). All software was accessed through the SBGrid consortium [34].

### Steady-state kinase activity assay

Kinase activity assays were carried out as described previously [19]. The Michaelis constant for ATP (*K*_M, ATP_) was determined in a 384-well assay plate (20 μl) by incubating purified phosphorylated (at 100 nM) or unphosphorylated (at 500 nM) MuSK TKD in the reaction buffer (50 mM HEPES, pH 7.5, 30 mM NaCl, 10 mM MgCl_2_, 0.2% (w/v) glycerol, 0.01% (v/v) Brij-35, and 1 mM DDT) containing 10 μM peptide substrate (AssayQuant, #Aqt0031) and a two-fold dilution series of ATP ranging from 1.56 to 3.2 mM for unphosphorylated MuSK or 0.98 to 2 mM for autophosphorylated MuSK. Reaction progress was monitored at the emission wavelength (λ_em_) of 480 nm for 4 h at 30°C with excitation wavelength (λ_ex_) of 360 nm on a BioTek Synergy 2 plate reader (Agilent). Blank fluorescence counts were subtracted from the sample counts to obtain relative fluorescence units (RFU). To convert RFU to the molar concentration of the phosphorylated peptide substrate, a standard curve was generated using a 1.5-fold dilution series of 100 μM Aqt0031 peptide substrate in the presence of 1 μM autophosphorylated MuSK^WT^ with the fluorescence monitored over 4 h (for complete phosphorylation). Initial velocities (*v*_0_) at different ATP concentrations were determined by inspection of progress curves during the steady-state phases (Supplementary Figure S2A). The *v*_0_ values were plotted against ATP concentrations and fitted to the MM equation to obtain *K*_M, ATP_ and the maximum velocity (*V*_max_) using GraphPad Prism. Similarly, Michaelis constants for the Aqt0031 peptide substrate (*K*_M, peptide_) and *k*_cat_ were determined by incubating 500 nM unphosphorylated or 100 nM autophosphorylated MuSK TKD with 1 mM ATP and a 1.5-fold dilution series of Aqt0031 ranging from 2.6 to 100 μM (Supplementary Figure S2C).

For calculating *k*_cat_ (*k*_cat_ = *V*_max_/[E]_active_), the concentration of active MuSK ([E]_active_) was determined by titration of a tight-binding kinase inhibitor, staurosporin (1.5-fold dilution series ranging 3.5 nM to 200 nM), in the presence of 1 mM ATP and 10 μM Aqt0031, using 100 nM autophosphorylated MuSK^WT^. The measured initial velocity (normalized to the initial velocity without the inhibitor) was plotted against staurosporine concentration and fitted to the equation *v*_i_/*v*_0_ = 100/1+[I]/IC_50_ using GraphPad Prism to obtain IC_50_ (Supplementary Figure S2D). Active [MuSK] was then determined based on the following assumption: for a tight-binding inhibitor, IC_50_ ∼ [E]_active_/2 + *K*_I, apparent_, where [E]_active_ = active enzyme concentration and *K*_I, apparent_ = apparent inhibition constant. For a tight-binding inhibitor, *K*_I, apparent_ becomes negligibly small so that IC_50_ ∼ [E]_active_/2. Based on this relationship, 99% of the purified MuSK TKD was determined to be active. All experiments were performed in technical triplicate and repeated using three separate protein preparations (*n* = 3) unless specified otherwise.

### Hydrogen–deuterium exchange and mass spectrometry

Freshly purified MuSK TKD at 0.6 mg/ml (15 μM) in 20 mM Tris pH 8.5, containing 150 mM NaCl, 1% (w/v) glycerol, and 1 mM TCEP (5 μl), was manually labeled by 20-fold dilution with D_2_O-buffer containing 20 mM HEPES (pD 7.4) and 100 mM NaCl at 25°C. The labeled sample was quenched at different time points (10, 60, 600, 3600, and 7200 s) by adding 100 μl of cold 400 mM glycine buffer containing 1 mM TCEP (at pH 2.3). Similarly, a fully deuterated sample was prepared by labeling the protein sample for 1 min with 20 mM HEPES (pD 7.4), 100 mM NaCl, in the presence of 8 M urea–d4 (Cambridge Isotope Laboratories, Inc.). Quenched samples were flash-frozen in liquid N_2_ and stored at −80°C until mass spectrometry analysis. Frozen samples were quickly thawed and manually injected onto an Enzymate BEH pepsin column (Waters) at 2°C, and the deuterium-labeled sample was digested for 3 min in solvent A (water + 0.1% formic acid) at 100 μl/min. Peptic peptides were trapped and separated using an Acquity UPLC BEH C18 pre-column (2.1 × 5 mm, 1.7 μm, Waters) and an Acquity UPLC BEH C18 column (1.0 × 100 mm, 1.7 μm, Waters), respectively, using a linear gradient from 5% to 40% (v/v) solvent B (acetonitrile + 0.1% formic acid) over 7 min at 40 μl/min. MS^e^ data were acquired using a Synapt G2-Si mass spectrometer (Waters) using a 0.4 s scan time and a ramp collision energy of 5 V to 10 V for LE and 15 V to 40 V for HE with continuous lock mass (Leu-Enk) for mass accuracy correction. Other instrument parameters were capillary voltage, 3 kV; cone voltage, 10 V; source offset, 80 V; source temperature, 100°C; desolvation temperature, 250°C; cone gas flow, 0.0 l/h; desolvation gas flow, 700 l/h; nebulizer gas, 6.5 bar; and MS scan range, 300–2000 m/z.

### HDX-MS data analysis

Peptides were sequenced using the ProteinLynx Global Server 3.03 (PLGS, Waters), and the deuterium uptake of each peptic peptide was determined using DynamX 3.0 (Waters). The deuterium uptake of all analyzed peptides presented in the present study is the average uptake of three biological samples with each biological repeat performed in technical triplicate. The percent exchange of each peptic peptide (%D) was calculated by the following equation:

$$\%\mathrm{Ex}=\frac{\left(m_{t}-m_{0}\right)}{\left(m_{f}-m_{0}\right)}\cdot 100,$$

where *m_t_* is the centroid mass of a peptic peptide at time, *t*; *m*_0_ is the centroid mass of a peptic peptide without deuterium labeling; and *m_f_* is the centroid mass of a peptic peptide for the fully deuterated standard sample. All data were collected and analyzed according to consensus HDX-MS guidelines [35]. The analyzed HDX-MS data are provided as a Supplemental Excel spreadsheet.

## Supplementary Material

Supplementary Figures S1-S8 and Table S1

## Data Availability

Crystal structures of human MuSK kinase domain presented in the present study have been deposited in the Protein Data Bank, with the accession PDB numbers 9SN0 [36] and 9SNU [37], respectively. Raw diffraction images were deposited at Zenodo (doi: 10.5281/zenodo.17086410). All HDX-MS data for WT and S752D MuSK KD were deposited in the PRIDE database [38] in RAW format under accession numbers PXD071785 and PXD072130, respectively.

## Open Access

Open access for this article was enabled by the participation of Yale University in an all-inclusive Read & Publish agreement with Portland Press and the Biochemical Society.

## Abbreviations

AChRs: acetylcholine receptors
A-loop: activation loop
CKs: casein kinases
CRD: cysteine-rich domain
ECR: extracellular region
GSK: glycogen synthase kinase
GRKs: G protein-coupled receptor kinases
HDX-MS: hydrogen–deuterium exchange and mass spectrometry
IgG-like: immunoglobulin G-like
JM: juxtamembrane
MM: Michaelis–Menten
MuSK: Muscle-Specific Kinase
MuSK^WT^: wild-type MuSK
MuSK^S751D^: S751D MuSK
NMJ: neuromuscular junction
PTB: phosphotyrosine-binding
RFU: relative fluorescence units
RTK: receptor tyrosine kinase
TKD: tyrosine kinase domain
WT: wild-type
