# Supplementary material for: An S752D activation loop mutation dynamically primes Muscle-Specific Kinase for activation
Source: Biochem J. 2026 Jun 24;483(7):1221–35. doi: 10.1042/BCJ20260159 (PMC13305817; doi:10.1042/BCJ20260159)
Supplement: Supplementary Figures S1-S8 and Table S1 [file BCJ-2026-0159_supp.pdf]

Table S1: The residue number and amino acid sequence for each peptide ID (accompanying table for Figure 4A)

Peptide ID #	Residues	Amino Acid Sequence
1	534-544	VTLTTLPSELL
2	537-543	TTLPSEL
3	537-544	TTLPSELL
4	544-554	LLDRLHPNPMY
5	555-565	QRMPLLLNPKL
6	566-573	LSLEYPRN
7	566-576	LSLEYPRNNIE
8	566-577	LSLEYPRNNIEY
9	577-586	YVRDIGEGAF
10	578-586	VRDIGEGAF
11	587-598	GRVFQARAPGLL
12	587-605	GRVFQARAPGLLPYEPFTM
13	608-619	VKMLKEEASADM
14	611-619	LKEEASADM
15	624-630	QREAALM
16	630-641	MAEFDNPNIVKL
17	630-645	MAEFDNPNIVKLLGVC
18	633-641	FDNPNIVKL
19	633-645	FDNPNIVKLLGVC
20	642-653	LGVCAVGKPMCL
21	642-654	LGVCAVGKPMCLL
22	646-653	AVGKPMCL
23	646-654	AVGKPMCLL
24	655-663	FEYMAYGDL
25	655-665	FEYMAYGDLNE
26	657-663	YMAYGDL
27	657-665	YMAYGDLNE
28	666-685	FLRSMSPHTVCSLSHSDLSM
29	667-679	LRSMSPHTVCSLS
30	667-683	LRSMSPHTVCSLSHSDL
31	667-685	LRSMSPHTVCSLSHSDLSM
32	677-685	SLSHSDLSM
33	678-685	LSHSDLSM
34	679-685	SHSDLSM
35	684-699	SMRAQVSSPGPPPLSC
36	686-699	RAQVSSPGPPPLSC
37	704-713	CIARQVAAGM
38	705-714	IARQVAAGMA
39	705-715	IARQVAAGMAY
40	716-732	LSERKFVHRDLATRNCL
41	738-743	VVKIAD
42	738-744	VVKIADF
43	738-746	VVKIADFGL
44	740-746	KIADFGL
45	756-775	YKANENDAIPIRWMPPESIF
46	776-785	YNRYTTESDV
47	793-809	WEIFSYGLQPYYGMAHE
48	814-827	YVRDGNILSCPENC

49	814-831	YVRDGNILSCPENCPVEL
50	815-831	VRDGNILSCPENCPVEL
51	835-849	MRLCWSKLPADRPSF
52	838-849	CWSKLPADRPSF
53	840-849	SKLPADRPSF
54	850-861	TSIHRILERMCE
55	862-869	RAEGTVSV

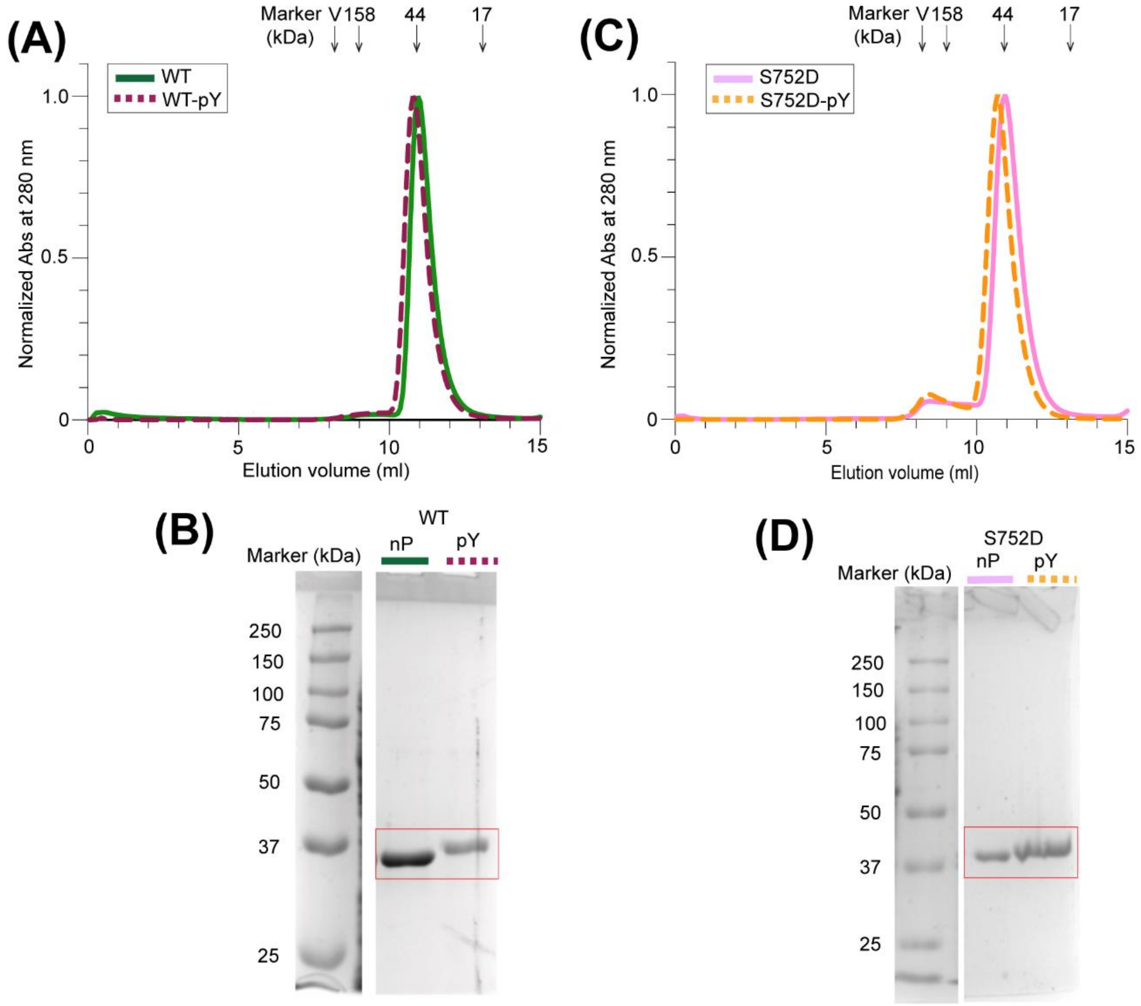

Supplementary Figure S1: Expression and purification of human MuSK kinase domain. Size exclusion chromatogram and SDS-PAGE of **(A-B)** wild-type (WT) and **(C-D)** S752D MuSK are shown. In (A) and (C), the elution volume and molecular weight of the size exclusion markers (Blue dextran (v) as a void volume marker; γ-globulin, 158 kDa; ovalbumin, 44 kDa; horse myoglobin, 17 kDa are indicated as black arrows. The dotted lines in each chromatogram show autophosphorylated (pY) wild-type or S752D. In (B,D), the size exclusion fractions of unphosphorylated (nP) and autophosphorylated (pY) wild-type and S752D are indicated in the red box.

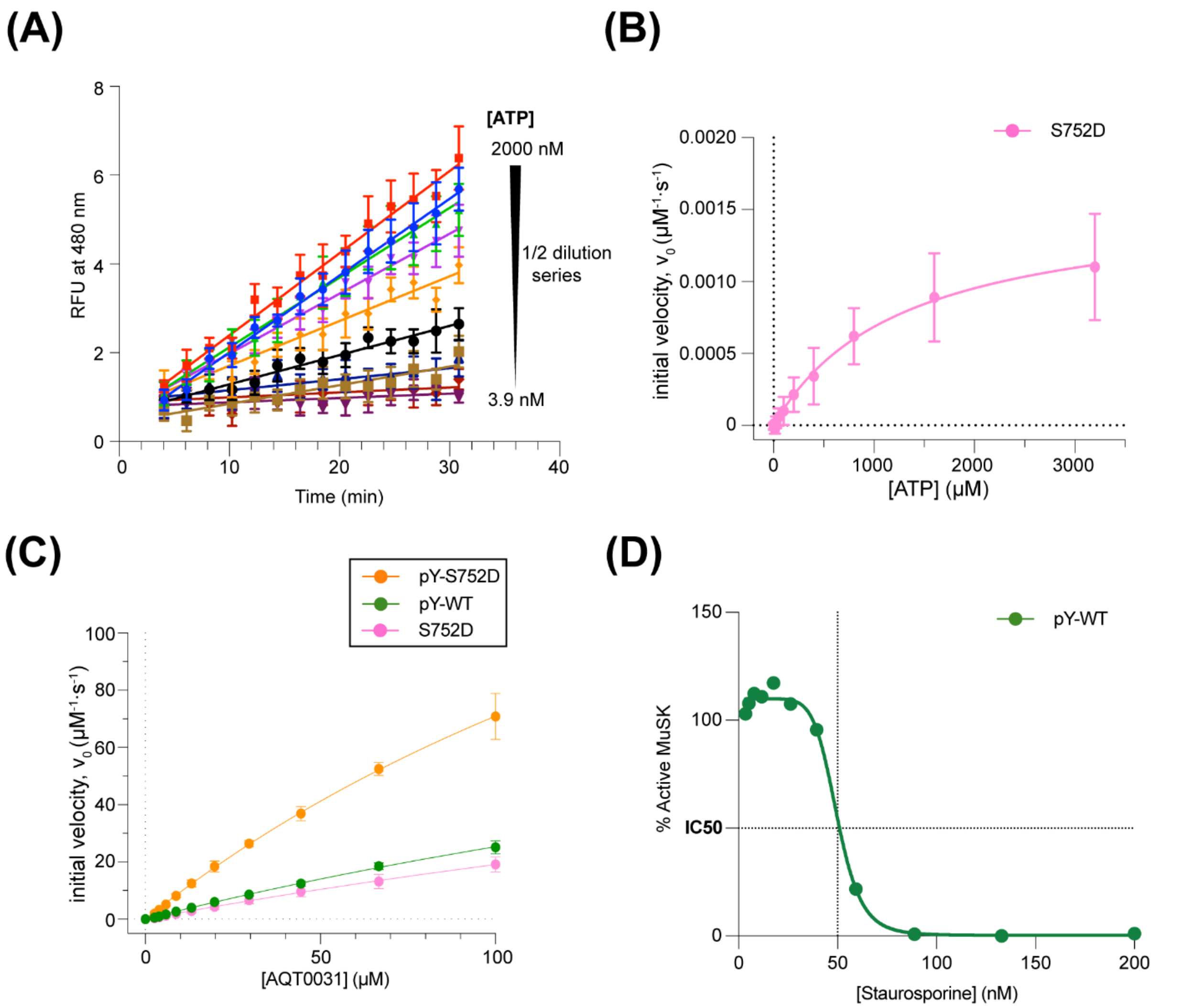

Supplementary Figure S2: Determination of steady-state kinetic parameters and active MuSK TKD concentration. (A) A representative raw progress curve for the determination of the Michaelis-Menten constant for ATP ($K_{M,\ ATP}$) is shown. Steady-state regions were fit to a linear equation to obtain the initial velocity at each ATP concentration, as shown. Relative fluorescence units (RFU) at 480 nm are converted to the molar concentration of phosphorylated peptide substrate as described in *the Methods*. Error bars here represent SD of three technical replicates of one experiment using a single protein preparation **(B)** Michaelis-Menten plots of unphosphorylated MuSK^{S752D} TKDs in the presence of 10 μM peptide substrate with MuSK concentration listed in *the Methods*. $K_{M,\ ATP}$ for MuSK^{WT} TKD could not be obtained due to the sub-saturating [ATP] used in our assay (See *Methods*). Error bars represent SD of three separate experiments using individual protein preparations. (**C**) A Michaelis-Menten plot for unphosphorylated S752D (pink) and *in vitro* autophosphorylated WT (green, pY-WT) and S752D (orange, pY-S752D) MuSK at different peptide substrate (AQT0031) concentration. Data were fit to the Michaelis-Menten equation to obtain $K_{M,\ ATP}$ as listed in Table 1. (**D**) Dose response curve obtained using 100 nM phosphorylated

$MuSK^{WT}$ TKD (green, pY-WT) in the presence of 10 μM peptide substrate and 1 mM ATP for determining active MuSK TKD concentration. Normalized initial velocity values (V_i/V_0) at different staurosporine concentrations were plotted and fit to the Langmuir isotherm to obtain an IC_{50} value (49.5 nM), suggesting that almost 100% of the TKD is active. The data show the average of three technical repeat experiments with one biological repeat (n=1).

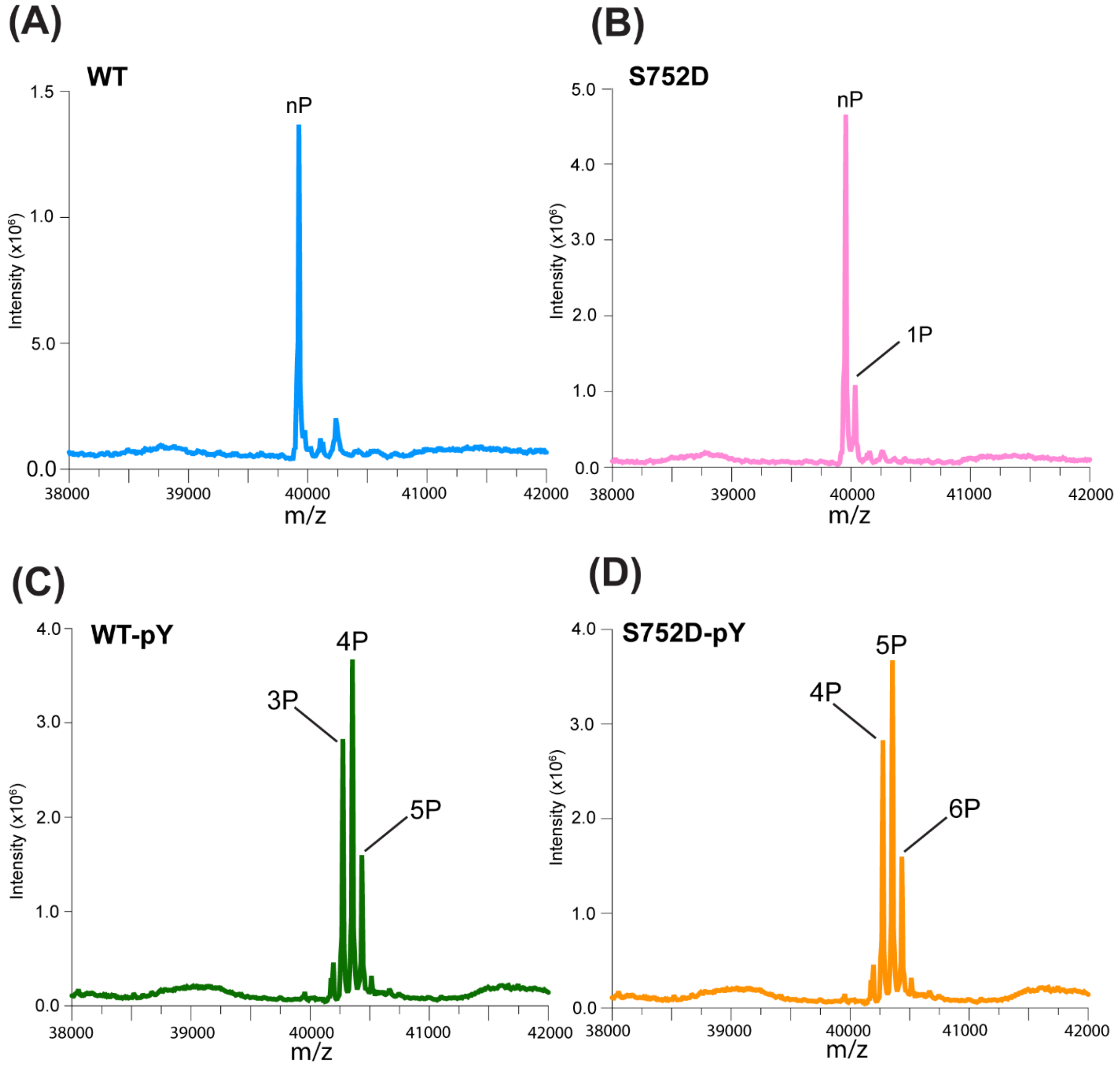

Supplementary Figure S3: Intact mass analysis of purified MuSK. Deconvoluted mass spectra of purified **(A)** unphosphorylated (nP) wild-type (WT) and **(B)** S752D MuSK kinase domains are shown. **(C-D)** Deconvoluted mass spectra of autophosphorylated (pY) (C) WT and (D) S752D are shown. The number of phosphorylations, as judged by 80 Dalton mass shift, is indicated. Table 2 lists the average mass of each protein population. In unphosphorylated S752D, ~5-10 % of a singly-phosphorylated protein (1P) was observed.

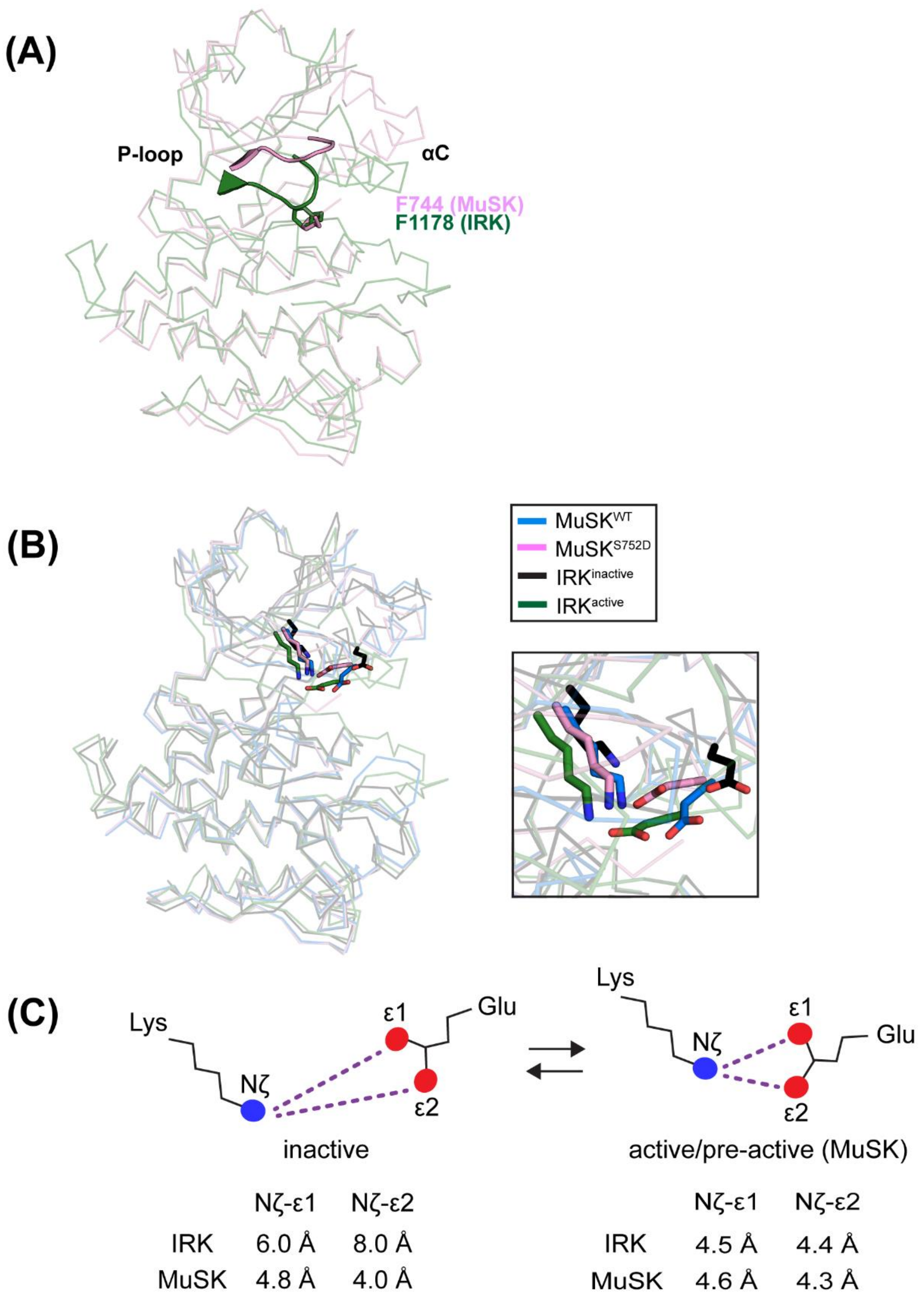

Supplementary Figure S4: MuSK^{S752D} is not in a fully active conformation. (A) Overlay of crystal structures of active IRK (green, PDB ID:1IR3) and MuSK^{S752D} (pink) with DFG phenylalanine in IRK (residue 1178 in Uniprot numbering) and MuSK (residue 744) shown as

green and pink sticks, respectively. P-loop in both structures are shown as thick ribbons. **(B)** Overlay of $MuSK^{WT}$, $MuSK^{S752D}$, IRK in active (PDB ID: 1IR3), and in inactive (PDB ID: 1IRK) crystal strutures. The β3-αC salt bridge pair in IRK (K1057-E1074 in Uniprot numbering) and MuSK (K609-E626) are colored as indicated and shown as sticks. (inset) a close-up view of the β3-αC salt bridge pair. **(C)** A summary of the β3-αC salt bridge pair distance in IRK and MuSK crystal structures.

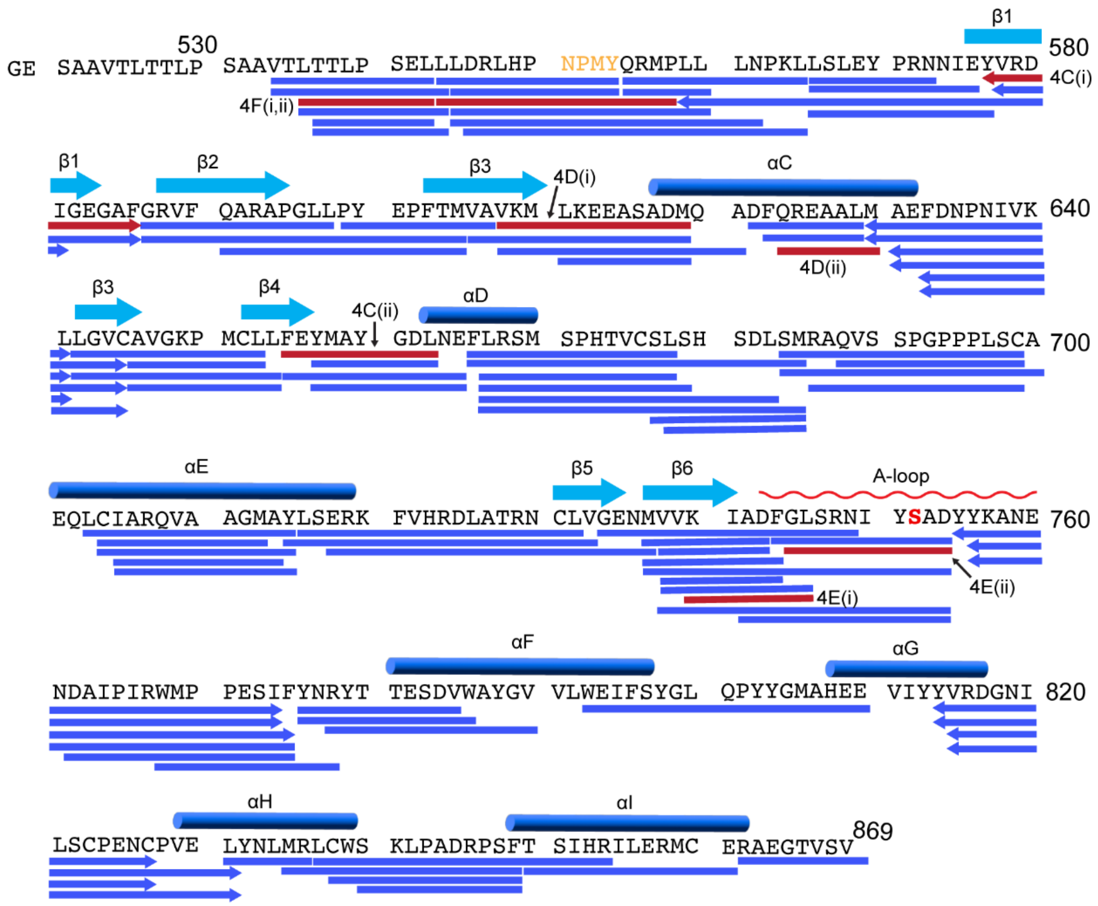

Supplementary Figure S5: peptide coverage for MuSK WT HDX-MS. The amino acid sequence of WT MuSK TKD is shown with the secondary structure regions above the sequence. Blue horizontal bars are analyzed peptides. Red bars indicate peptides used to present their percent exchange in Figure 4.

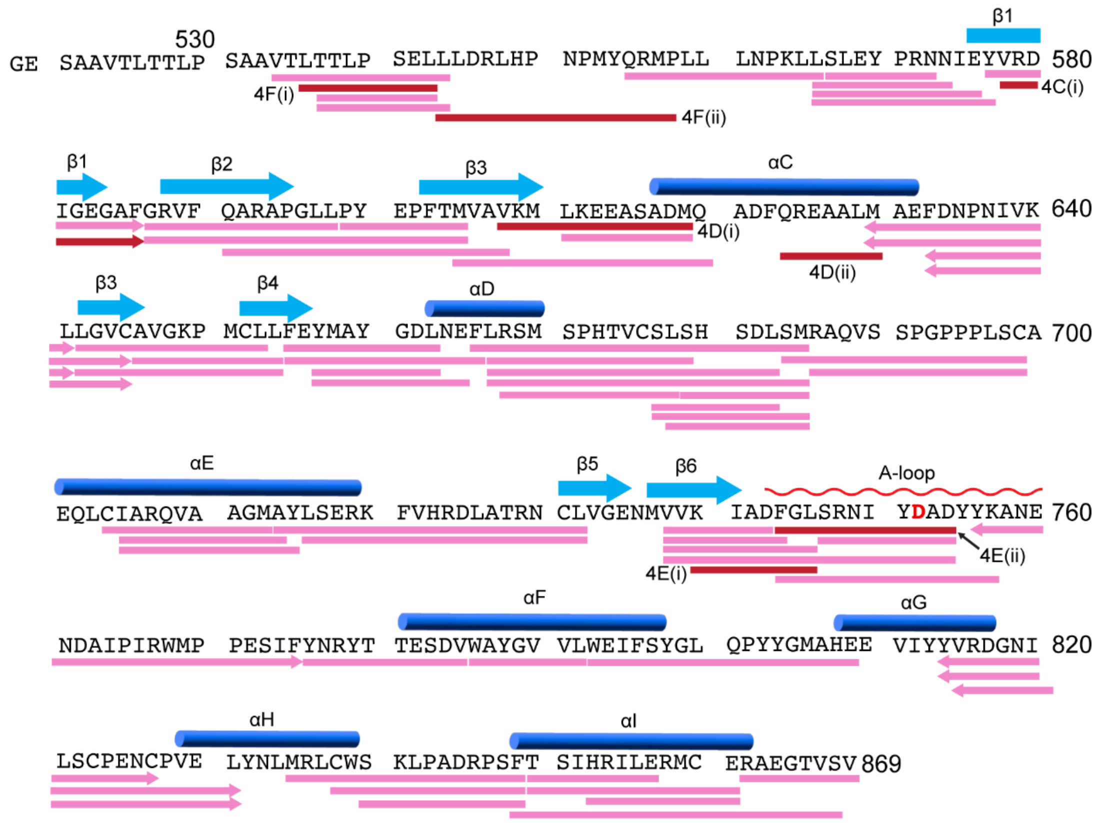

Supplementary Figure S6: peptide coverage for MuSK S752D HDX-MS. The amino acid sequence of MuSK S752D TKD is shown with the secondary structure regions above the sequence. Pink horizontal bars are analyzed peptides. Red bars indicate peptides used to present their percent exchange in Figure 4.

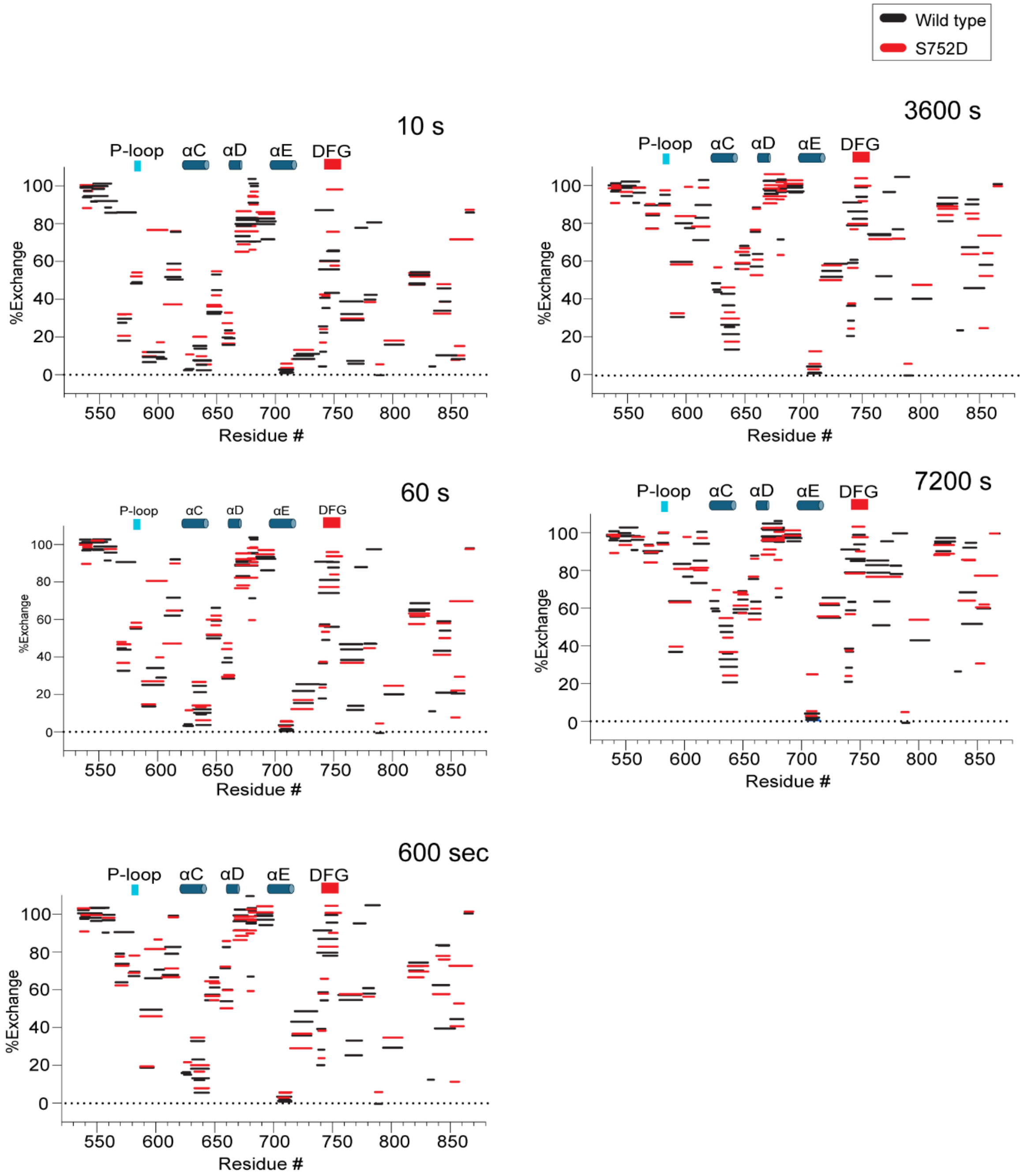

Supplementary Figure S7: S752D mutation disrupt autoinhibition by increasing A-loop dynamics. Woods plot of MuSK^{WT} (black) and MuSK^{S752D} (red) at the indicated labeling time points. All analyzed peptides are shown. The percent exchange of each peptide is shown as colored horizontal bars on the y-axis with the x-axis indicating the residue number. The position of the P-loop, the N-lobe helices, including αC, αD, and αE, and the DFG motif is indicated at the top of the figure. The average percent exchange data from 3 biological repeat experiments (n=3) with 3 technical repeats (3/n) are shown.

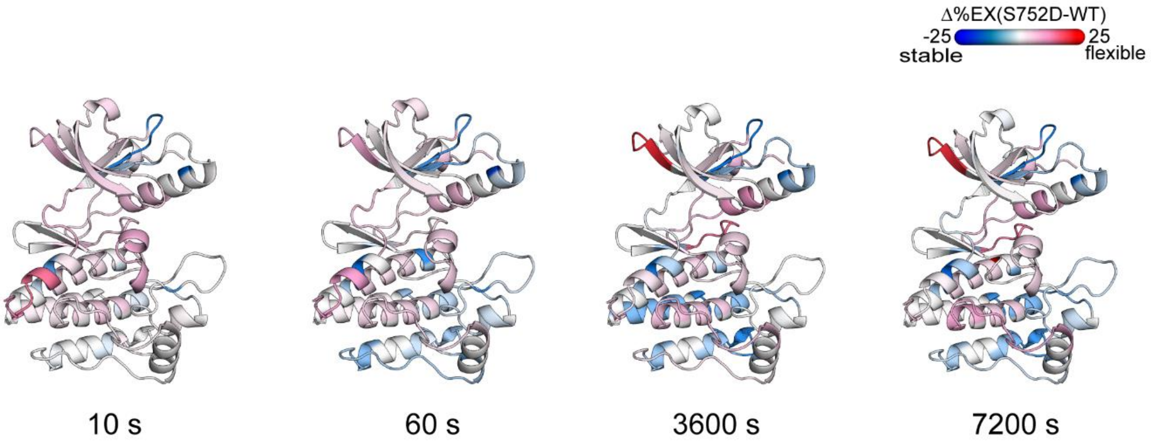

Supplementary Figure S8: The S752D mutation increases structural flexibility. The percent exchange differences between MuSK^{S752D} and MuSK^{WT} (Δ%EX) are color-coded as shown and mapped onto a crystal structure of human MuSK^{WT} TKD. Red regions become more flexible with S752D mutation compared to WT. Blue regions are stabilized regions with S752D mutation. Dark grey regions show missing peptide regions.
